# QServer: A Biclustering Server for Prediction and Assessment of Co-Expressed Gene Clusters

**DOI:** 10.1371/journal.pone.0032660

**Published:** 2012-03-05

**Authors:** Fengfeng Zhou, Qin Ma, Guojun Li, Ying Xu

**Affiliations:** 1 Research Center for Biomedical Information Technology, Institute of Biomedical and Health Engineering, Shenzhen Institutes of Advanced Technology, Chinese Academy of Sciences, Shenzhen, People's Republic of China; 2 Computational Systems Biology Laboratory, Department of Biochemistry and Molecular Biology, Institute of Bioinformatics, BioEnergy Science Center (BESC), University of Georgia, Athens, Georgia, United States of America; 3 School of Mathematics, Shandong University, Jinan, China; 4 College of Computer Science and Technology, Jilin University, Changchun, China; Wayne State University, United States of America

## Abstract

**Background:**

Biclustering is a powerful technique for identification of co-expressed gene groups under any (unspecified) substantial subset of given experimental conditions, which can be used for elucidation of transcriptionally co-regulated genes.

**Results:**

We have previously developed a biclustering algorithm, QUBIC, which can solve more general biclustering problems than previous biclustering algorithms. To fully utilize the analysis power the algorithm provides, we have developed a web server, QServer, for prediction, computational validation and analyses of co-expressed gene clusters. Specifically, the QServer has the following capabilities in addition to biclustering by QUBIC: (i) prediction and assessment of conserved *cis* regulatory motifs in promoter sequences of the predicted co-expressed genes; (ii) functional enrichment analyses of the predicted co-expressed gene clusters using Gene Ontology (GO) terms, and (iii) visualization capabilities in support of interactive biclustering analyses. QServer supports the biclustering and functional analysis for a wide range of organisms, including human, mouse, *Arabidopsis*, bacteria and archaea, whose underlying genome database will be continuously updated.

**Conclusion:**

We believe that QServer provides an easy-to-use and highly effective platform useful for hypothesis formulation and testing related to transcription co-regulation.

## Introduction

Microarray gene expression chips provide a powerful tool for studying transcription and transcriptional regulation at a systems level. Using this technique, scientists have been generating large quantities of gene-expression data for various organisms under a variety of experimental conditions, aiming to collect sufficient gene-expression data for elucidation of local as well as global transcription regulation networks in these organisms. One of the frequently used analysis techniques of such data is *biclustering* for identifying co-expressed genes under some (to-be-identified) subsets of the specified experimental conditions. Mathematically the problem can be modeled as finding all statistically significant sub-matrices of a representing matrix of the expression levels of genes (rows) of an organism collected under multiple conditions (columns), each of which exhibits (row-wise) similarities or correlations. Computationally this is a very challenging problem since it involves (implicitly) going through all combinations of the subsets of the genes and the subsets of the conditions. Morgan and Sonquist [1] developed the first algorithm for solving a biclustering problem though the word “biclustering” was first proposed by Cheng and Church [2]. Because of the potential applications of the biclustering strategy as well as the challenging nature in solving the problem computationally, a number of research groups have proposed various algorithmic techniques for solving the problem [3], [4], [5], [6], [7] with varying degrees of success and usefulness.

We have recently developed a biclustering algorithm QUBIC, and demonstrated its superior performance when compared to other algorithms on various benchmark sets [8]. While QUBIC can be installed and used as a stand-alone prediction package, we found that the tool becomes more useful when applied in conjunction with other tools, particularly prediction tools of *cis* regulatory motifs and for functional enrichment analyses. Based on this consideration, we developed the QServer, which integrates these capabilities with QUBIC and is packaged as an easy-to-use software tool. While there have been a number of published biclustering algorithms, to the best of our knowledge, there is only a few biclustering prediction servers on the Internet, namely, a Gibbs sampling-based method GEMS [9] and a non-negative factorization method bioNMF [10]. Some servers, e.g. BiCAT [11] and BiGGEsTS [12], provide only downloadable versions of their executable programs where users cannot run jobs directly on the servers. Compared to these prediction servers, QServer provides a substantially richer set of capabilities and a user-friendly environment on top of its superior biclustering performance.

## Materials and Methods

### Biclustering analysis using QUBIC

The algorithm of QUBIC [8] employs a graph-theoretic approach to solve the biclustering problem. It employs a preprocessing step to discretize the continuous values of gene expression levels in a qualitative way, with the detailed discretization process determined by two parameters *q* and *r*, where *q* is the proportion of the affected expression data under all the conditions for each gene and *r* represents the rank of the regulating conditions detected by the parameter *q*. The biclustering problem is modeled as finding dense subgraphs with specific properties in a weighted graph defined over the input data, with genes represented as vertices of the graph and edges connecting gene pairs with similar expression patterns (with similarity above a predefined threshold) whose weights reflect the levels of similarity.

The program has a number of adjustable parameters including (i) a parameter that controls the level of discretization of the continuous values of the gene expression data, (ii) the minimum level of consistency required among rows and columns of the to-be-identified biclusters, (iii) the level of maximum allowed overlaps between two to-be-identified biclusters and (iv) the upper limit on the number of biclusters to be output by the program. The general guideline is to use the default parameters of the program, which have been optimized on a few large training datasets [8]. Clearly different combinations of parameters may lead to different results, e.g. a smaller value of parameter (iii) can generate fewer biclusters.

On a dataset with tens of thousands of genes under up to thousands of conditions, it typically takes QUBIC a few minutes of the CPU time on one desktop computer to find most of the statistically significant biclusters. Thus QServer has a wider application range than other biclustering web-servers.

### Motif finding in promoters of co-expressed genes

The predicted clusters of co-expressed genes may represent genes that are transcriptionally co-regulated [13]. The QServer provides a capability for computationally validating that by predicting conserved *cis* regulatory motifs among the promoter sequences which are automatically extracted from the upstream sequences (the default value is 300 bps long) of the co-expressed genes. Currently QServer provides the option of two motif prediction programs, BOBRO [14] developed by our group and the popular MEME program [15]. Both programs attempt to find conserved sequences among a set of given promoter sequences using different strategies, and both programs provide a statistical significance score for each predicted motif. BOBRO has a number of distinct capabilities as detailed in [14]. Clearly when a group of predicted co-expressed genes are found to have conserved motifs in their promoter sequences, it provides strong evidence that these genes might be indeed transcriptionally co-regulated; otherwise, the user may want to check if the predicted co-expressed gene cluster has low enough statistical significance.

### Functional enrichment analysis

For each cluster of predicted co-expressed genes, QServer provides a capability for functional enrichment analysis based on GO classification [16]. Such a capability allows a user to link an identified co-expressed gene cluster to known biological pathways. Specifically, we have implemented the following calculation. Consider a GO term, GOF. We check if the term is enriched in a bicluster, BCF, compared against the background gene distribution, i.e. the whole genome, across different GO functional classes. The *enrichment ratio* (ER) of GOF is defined to describe whether GOF is over- (ER>1) or under- (ER<1) represented in bicluster BCF. Assuming the null hypothesis that genes with GOF are uniform-randomly distributed over the whole genome, we calculated the P-value and ER as similar in [17], [18]. An over-represented GO term with P-values< = 0.05 rejects the above null hypothesis.

### Using QServer

From the “BiCluster” page, a user can input a gene-expression matrix through the text box. QServer uses the popular gene-expression matrix format, with rows representing genes/probes and columns representing samples/conditions, as described in [8]. The file title and other comment lines should start with “#”. The user can normalize the expression matrix using any poplar algorithms such as RMA [19], MAS5 [20] or Plier [21] provided in the Affymetrix Power Tools.

After copying and pasting the input matrix into the text box, the user can start the calculation of biclusters in the matrix by clicking “Submit”, which returns with a JobID. The analysis result can be later retrieved using the JobID through the “Retrieval” page (Figure S2).

**Figure 1:**
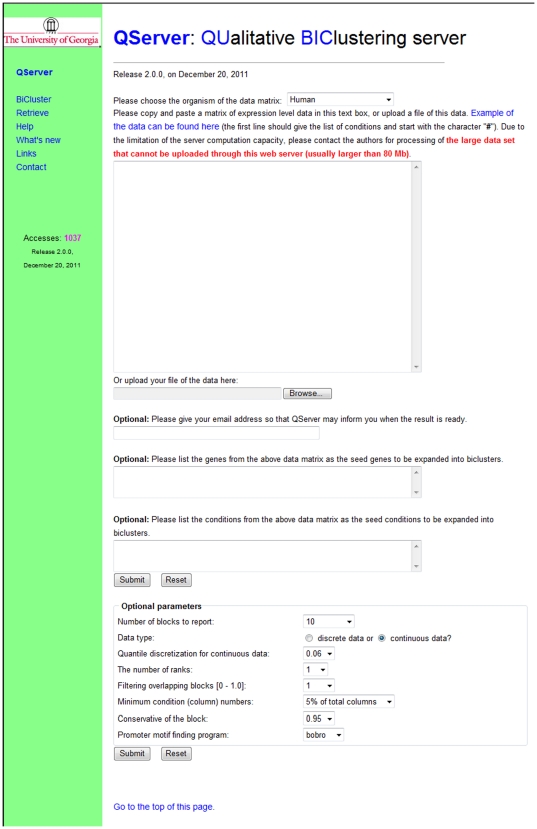
BiCluster page. The “BiCluster” page is the interface for the user to provide an expression matrix and to change the default values of the optional parameters.

The user can display specific biclusters by searching the rows and columns with keywords specified in two text boxes above “Optional parameters” (see Figure 1). All the entries in “Optional parameters” can be changed by the user for specific applications, as shown in Figure 1. We generally suggest that the user uses the default parameters of the system. As to how to adjust parameters for different applications, the user can find the details from the “Help” page.

### Data sources

The QServer can do biclustering analyses for any matrix, including non-biological data, and plot heat maps for the calculated biclusters. A number of biological databases are available in the public domain, each of which may use different naming conventions for genes in its database. To deal with this issue, we have collected these naming conventions from multiple sources so that QServer can automatically detect the naming system used in a given data matrix. Overall we collected all the genome sequences and the gene annotations from the NCBI Genome database, along with three gene/protein naming systems, i.e. GI, locus and refseq. The detailed information of the versions and the release dates are listed in Table S1. The UniProtKB names of the encoded proteins were also retrieved from UniProtKB [22]. The GO and IPR domain annotations of the encoded proteins were collected from Integr8 [23], [24]. We retrieved the probe names of the two most widely used human array platforms (platform IDs GPL96 and GPL570) from the Gene Expression Omnibus (GEO) database [25]. The TAIR gene names of *A. thaliana* were retrieved from *The Arabidopsis Information Research* (TAIR) database [26]. The probe names of *E. coli* K12 MG1655 were collected from the Many Microbe Microarrays Database (M3D) [27]. If a user's target genome is not in the above list, QServer will only do the biclustering and heat map plotting.

### Result retrieval

A user can retrieve the result of a previously submitted job using the JobID from the “Retrieve” page and may click the button “Example Job” to supply the JobID of the example data set, QUBIC_4b1c16f8300a48.01510801, and click the button “Submit” to get the result as a practice. A heat map of this expression matrix and a summary table of the identified biclusters are given in the top of the page using an in-house Perl script and the Perl GD library by Lincoln Stein at http://search.cpan.org/~lds/GD-2.44/, and a user may check the detailed information of a bicluster by clicking on “Details” in the row representing the bicluster in the table. For each identified bicluster, a sub-matrix is displayed as a table, in which the up- or down-regulation of a gene is represented as 1 or -1, respectively, with 0 for no change. The heat map for each bicluster is highlighted using a surrounding green box by moving the rows and columns of this bicluster to the top left corner of the matrix. Since biclusters may overlap with each other, it may not always be possible to visualize all identified biclusters simultaneously.

The statistically enriched GO terms and identified promoter motifs are listed next to each identified bicluster (sub-matrix). We also provide a compressed version of all the identified biclusters for each job for downloading from the “Retrieve” page, so the user can do further analyses of the data by themselves.

### Other information

The most frequently asked questions with their answers and a detailed user manual can be found in the “Help” page. A useful tool, *Matrix Maker 1.0*, was also provided on the “Help” page to generate an expression matrix in case a user may have only the raw .CEL file, supported by Affymetrix Power Tools. Furthermore, a comprehensive list of available utility biclustering tools is provided in the “Links” page.

### Availability and Future Directions

QServer is freely available at http://csbl.bmb.uga.edu/publications/materials/ffzhou/QServer/. The source code of QUBIC can be downloaded from http://csbl.bmb.uga.edu/~maqin/bicluster/qubic1.0.tar.gz. We will provide commonly used normalization algorithms in our server, so that the users may directly provide the raw data matrix, and test the hypothesis using different normalization algorithms.

## Results

### Summary of the QServer functionalities

**Figure 2:**
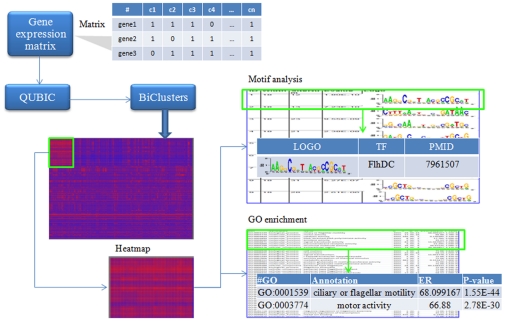
Using QServer. An example data of using QServer.

QServer has three modules for analyzing the input matrix. Firstly, the input matrix will be subject to biclustering analysis using QUBIC [8]. *cis* regulatory motifs were identified (the second module) using a motif finding tool, in the promoter regions of the genes in each bicluster. We provide two options for a user to choose, MEME [15] or BOBRO [14]. The detailed information of each identified motif, including its *p-value* and the logo plot, is also provided (see Figure 2). The third module is for identifying enriched GO categories [16] among genes in each bicluster. In addition, an example expression matrix of *E. coli K12* MG1655 [27] was included for demonstration purpose of the whole process of using the QServer (see Table S2). The user may find the details of the QUBIC algorithm, including both strengths and weakness, in [8] as compared with the other biclustering algorithms.

### QServer interface

The front page of the QServer (Figure S1) provides an introduction to the underlying biclustering algorithm along with a pull-down menu that supports the following functions: (a) biclustering by QUBIC through the “BiCluster” page (Figure 1); (b) result retrieval through the “Retrieve” page (Figure S2) and (c) a detailed user manual through the “Help” page (Figure S3). In addition, some useful links to gene-expression data analysis and biclustering servers can also be found through the “Links” page.

### An example of using QServer

**Figure 3:**
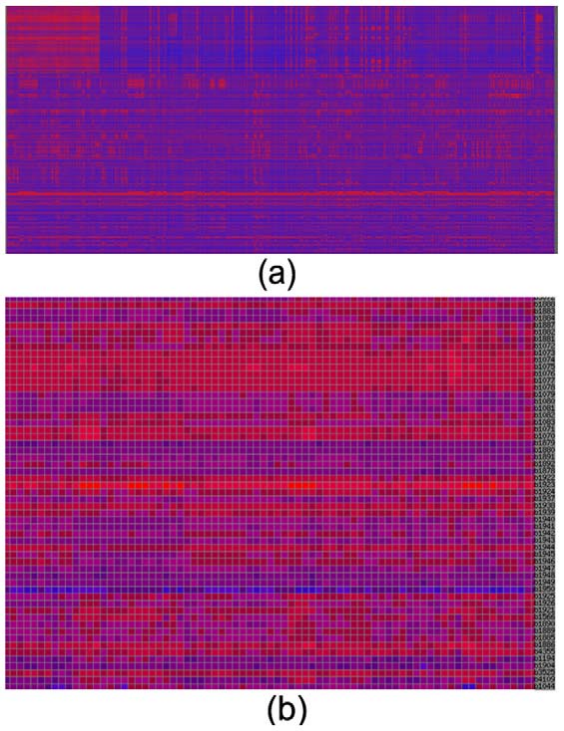
Heat map. (a) A complete version of the heat map of the example data in Figure 2; and (b) the heat map of the bicluster BC000 in the top-left corner of (a).

We now use a data matrix on *E. coli* as an example to illustrate using the QServer for biological knowledge discovery. We downloaded the whole data set of the M3D database (version 4 build 6) [27], and chose randomly 211 genes (rows) across 466 conditions (columns) as the example data matrix. The list of 211 genes is given in the Supplementary Materials. This matrix was entered through the “BiCluster*”* interface and the returned job id is QUBIC_4c8653d46110c3.88634327. After this job was finished, the result of the job can be retrieved through the *Retrieve* interface using the aforementioned job id. As an example, we carried out detailed analyses of the largest one (BC000) of the 36 identified biclusters. As shown in Figure 2, BC000 consists of 24 genes annotated to be in GO:0001539 (ciliary or flagellar motility) and 17 genes in GO: 0003774 (motor activity). The complete version of BC000's heat map can be found in Figure 3. The P*-values* of the enrichment of the two GO categories by these genes are 3.1e-46 and 2.3e-32, respectively. In addition, QServer identified the binding motif of the transcription factor FlhDC, which activates the flagellar class II operons [28]. Our data for the bicluster BC000 suggests that the flagellar-based motility is activated under the conditions in the columns of BC000.

## Discussion

Biclusters generated by a typical biclustering algorithm are not informative enough for a biologist to formulate hypothesis. It's generally requires additional information. We have implemented the most commonly used functionalities, including the heat map, enriched GO terms and promoter motif finding for the identified biclusters. We believe QServer will greatly facilitate non-trivial data analysis, discoveries and hypothesis formulation by its users through analyzing their large scale microarray data in an interactive fashion.

## Supporting Information

Figure S1 **Front Page.** An introduction and a pull-down menu.
(TIF)

Figure S2 **Retrieve page.** Detailed results including biclusters with heat map, GO enrichment and motif analysis can be retrieved from this page using a JobID.
(TIF)

Figure S3 **Help page.** A detailed user manual of how to use QServer and information of how to generate the input data matrix.
(TIF)

Table S1 **Raw data information.** Detailed information of the versions and release dates of the data source on QServer.
(PDF)

Table S2 **Example gene list.** 211 genes were used in the example data set.
(PDF)

## References

[1] Morgan JN, Sonquist JA (1963). Problems in the analysis of survey data, and proposal.. Journal of the American Statistical Association.

[2] Cheng Y, Church GM (2000). Biclustering of expression data.. Proc Int Conf Intell Syst Mol Biol.

[3] Hochreiter S, Bodenhofer U, Heusel M, Mayr A, Mitterecker A (2010). FABIA: factor analysis for bicluster acquisition.. Bioinformatics.

[4] Huttenhower C, Flamholz AI, Landis JN, Sahi S, Myers CL (2007). Nearest Neighbor Networks: clustering expression data based on gene neighborhoods.. BMC Bioinformatics.

[5] Madeira SC, Oliveira AL (2009). A polynomial time biclustering algorithm for finding approximate expression patterns in gene expression time series.. Algorithms Mol Biol.

[6] Prelic A, Bleuler S, Zimmermann P, Wille A, Buhlmann P (2006). A systematic comparison and evaluation of biclustering methods for gene expression data.. Bioinformatics.

[7] Waltman P, Kacmarczyk T, Bate AR, Kearns DB, Reiss DJ (2010). Multi-species integrative biclustering.. Genome Biol.

[8] Li G, Ma Q, Tang H, Paterson AH, Xu Y (2009). QUBIC: a qualitative biclustering algorithm for analyses of gene expression data.. Nucleic Acids Res.

[9] Wu CJ, Kasif S (2005). GEMS: a web server for biclustering analysis of expression data.. Nucleic Acids Res.

[10] Mejia-Roa E, Carmona-Saez P, Nogales R, Vicente C, Vazquez M (2008). bioNMF: a web-based tool for nonnegative matrix factorization in biology.. Nucleic Acids Res.

[11] Barkow S, Bleuler S, Prelic A, Zimmermann P, Zitzler E (2006). BicAT: a biclustering analysis toolbox.. Bioinformatics.

[12] Goncalves JP, Madeira SC, Oliveira AL (2009). BiGGEsTS: integrated environment for biclustering analysis of time series gene expression data.. BMC Res Notes.

[13] Werner T (2001). Cluster analysis and promoter modelling as bioinformatics tools for the identification of target genes from expression array data.. Pharmacogenomics.

[14] Li G, Liu B, Ma Q, Xu Y (2011). A new framework for identifying cis-regulatory motifs in prokaryotes.. Nucleic Acids Res.

[15] Bailey TL, Boden M, Buske FA, Frith M, Grant CE (2009). MEME SUITE: tools for motif discovery and searching.. Nucleic Acids Res.

[16] Gene Ontology Consortium (2010). The Gene Ontology in 2010: extensions and refinements.. Nucleic Acids Res.

[17] Zhou F, Olman V, Xu Y (2008). Insertion Sequences show diverse recent activities in Cyanobacteria and Archaea.. BMC Genomics.

[18] Zhou F, Xue Y, Lu H, Chen G, Yao X (2005). A genome-wide analysis of sumoylation-related biological processes and functions in human nucleus.. FEBS Lett.

[19] Irizarry RA, Bolstad BM, Collin F, Cope LM, Hobbs B (2003). Summaries of Affymetrix GeneChip probe level data.. Nucleic Acids Res.

[20] Liu WM, Mei R, Di X, Ryder TB, Hubbell E (2002). Analysis of high density expression microarrays with signed-rank call algorithms.. Bioinformatics.

[21] Seo J, Hoffman EP (2006). Probe set algorithms: is there a rational best bet?. BMC Bioinformatics.

[22] The UniProt Consortium (2009). The Universal Protein Resource (UniProt) in 2010.. Nucleic Acids Res.

[23] Kersey P, Bower L, Morris L, Horne A, Petryszak R (2005). Integr8 and Genome Reviews: integrated views of complete genomes and proteomes.. Nucleic Acids Res.

[24] Pruess M, Kersey P, Apweiler R (2005). The Integr8 project–a resource for genomic and proteomic data.. In Silico Biol.

[25] Barrett T, Troup DB, Wilhite SE, Ledoux P, Rudnev D (2009). NCBI GEO: archive for high-throughput functional genomic data.. Nucleic Acids Res.

[26] Poole RL (2007). The TAIR database.. Methods Mol Biol.

[27] Faith JJ, Driscoll ME, Fusaro VA, Cosgrove EJ, Hayete B (2008). Many Microbe Microarrays Database: uniformly normalized Affymetrix compendia with structured experimental metadata.. Nucleic Acids Res.

[28] Liu X, Matsumura P (1994). The FlhD/FlhC complex, a transcriptional activator of the Escherichia coli flagellar class II operons.. J Bacteriol.

