# Supplementary figures and images for: QServer: A Biclustering Server for Prediction and Assessment of Co-Expressed Gene Clusters
Source: PLoS One. 2012 Mar 5;7(3):e32660. doi: 10.1371/journal.pone.0032660 (PMC3293860; doi:10.1371/journal.pone.0032660)

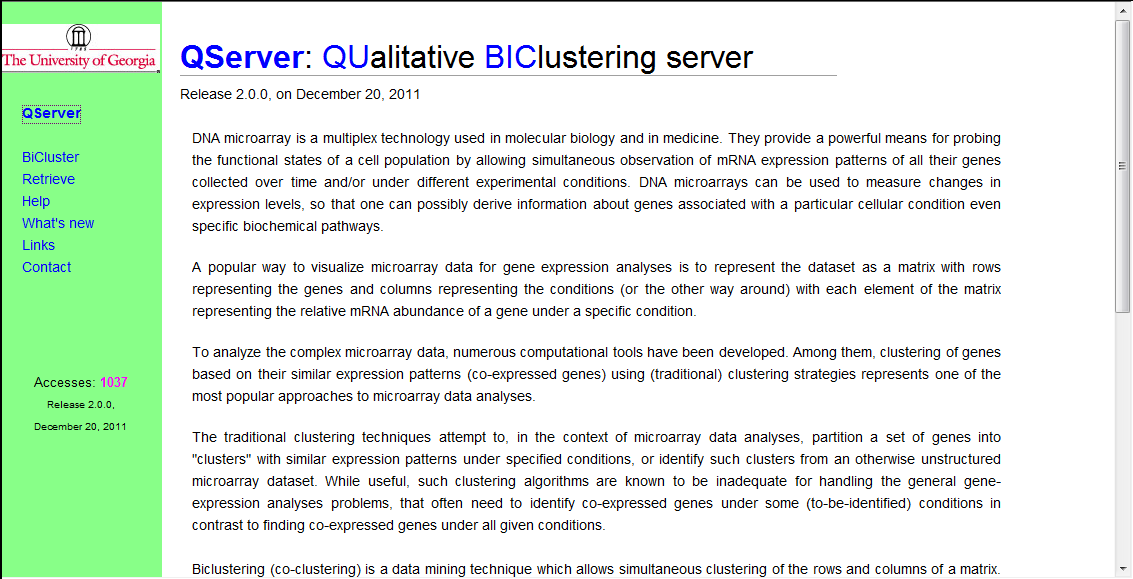

Supplement: Figure S1 — Front Page. An introduction and a pull-down menu. (TIF) [file pone.0032660.s001.tif]

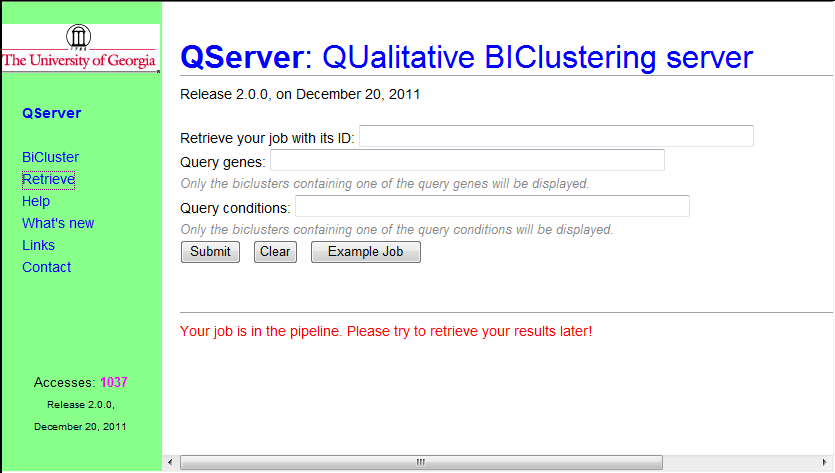

Supplement: Figure S2 — Retrieve page. Detailed results including biclusters with heat map, GO enrichment and motif analysis can be retrieved from this page using a JobID. (TIF) [file pone.0032660.s002.tif]

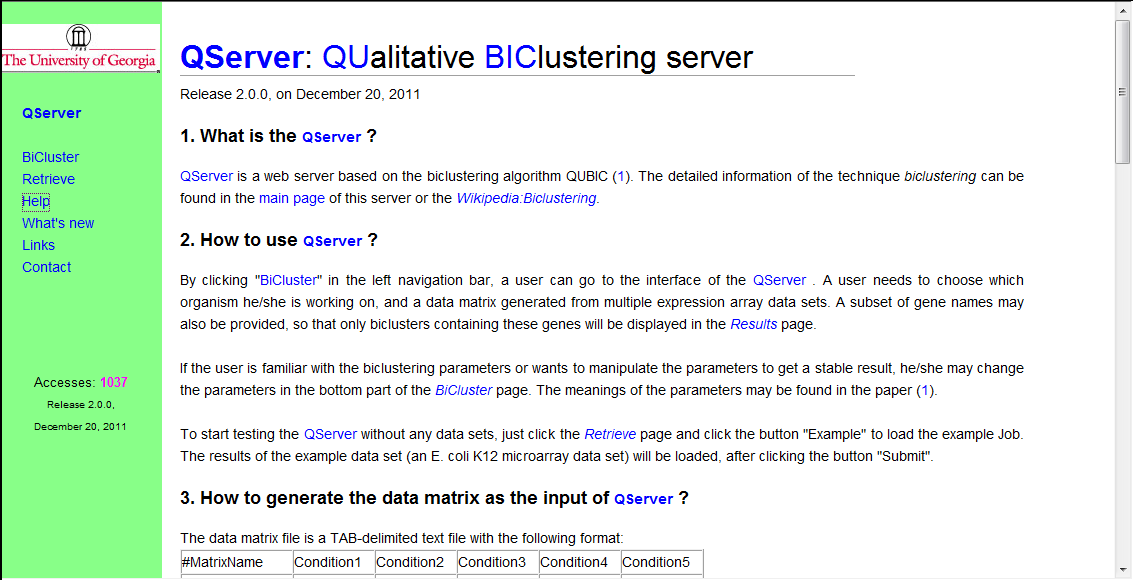

Supplement: Figure S3 — Help page. A detailed user manual of how to use QServer and information of how to generate the input data matrix. (TIF) [file pone.0032660.s003.tif]
