# Supplementary material for: QServer: A Biclustering Server for Prediction and Assessment of Co-Expressed Gene Clusters
Source: PLoS One. 2012 Mar 5;7(3):e32660. doi: 10.1371/journal.pone.0032660 (PMC3293860; doi:10.1371/journal.pone.0032660)
Supplement: Table S1 — Raw data information. Detailed information of the versions and release dates of the data source on QServer. (PDF) [file pone.0032660.s004.pdf]

Table S1: Detailed information of the versions and release dates of the data source on QServer.

Species	Genome sequences	GI	locus	refseq	uniport	GO	IPR	probe_ID	TAIR
Bucillus subtilis	8/24/09	8/24/09	8/24/09	8/24/09	9/21/09	8/29/09	8/29/09		
Escherichia coli K12 MG1655	8/24/09	8/24/09	8/24/09	8/24/09	9/21/09	8/29/09	8/29/09	v4_Build_5	
Arabidopsis thaliana	11/12/07	11/12/07	9/22/09	11/12/07	9/21/09	8/29/09	8/29/09	10/13/05	6/20/09
Mus musculus	6/12/09	6/12/09	6/12/09	6/12/09	9/21/09	8/29/09	8/29/09		
Homo sapiens	10/7/08	10/7/08	10/7/08	10/7/08	9/21/09	8/29/09	8/29/09	U133A: 04/28/09; U133_plus2: 06/30/09	
