# Supplementary material for: QServer: A Biclustering Server for Prediction and Assessment of Co-Expressed Gene Clusters
Source: PLoS One. 2012 Mar 5;7(3):e32660. doi: 10.1371/journal.pone.0032660 (PMC3293860; doi:10.1371/journal.pone.0032660)
Supplement: Table S2 — Example gene list. 211 genes were used in the example data set. (PDF) [file pone.0032660.s005.pdf]

## Table S2: Example gene list (containing 211 genes)

b3128, b1283, b1923, b2060, b1944, b1076, b2148, b0695, b1725, b1885, b3158, b1904, b2728, b3117, b3365, b1946, b2423, b4380, b2752, b3605, b1080, b4154, b1072, b1044, b2150, b1887, b2208, b1881, b3573, b2207, b2744, b4069, b3525, b2486, b3544, b2997, b0894, b1883, b3225, b2770, b0307, b3223, b2750, b4067, b4034, b0999, b2431, b2059, b1566, b2980, b0306, b1891, b3999, b1889, b3543, b3020, b3126, b2434, b2126, b1415, b3479, b0241, b3124, b4123, b4032, b3125, b0512, b4151, b2424, b0297, b2343, b1391, b1987, b1941, b1498, b1010, b2746, b1297, b0720, b3072, b2406, b0953, b1922, b2514, b4070, b3342, b1938, b1884, b2359, b1879, b2976, b3540, b2957, b2643, b4036, b1661, b1948, b0422, b1256, b1224, b2788, b2727, b4379, b1926, b1074, b2960, b1940, b1079, b0549, b3159, b0346, b4153, b1895, b2751, b2762, b1945, b1724, b3846, b1943, b1949, b1083, b2844, b0598, b0550, b0042, b2358, b1924, b1921, b1892, b2726, b1886, b0487, b2414, b3127, b3221, b1075, b2206, b1326, b4207, b3604, b4068, b1757, b2202, b2365, b2458, b1081, b2149, b1071, b1077, b4261, b1937, b4109, b2764, b3476, b1880, b3416, b0331, b0621, b1882, b1942, b4071, b3603, b3222, b2383, b1078, b1888, b4355, b3845, b2745, b1890, b0587, b1939, b3224, b2425, b2310, b1070, b1925, b0651, b3366, b4033, b1194, b1082, b1878, b1950, b4035, b3296, b3478, b1223, b0904, b4152, b4037, b4557, b2729, b2662, b1073, b2108, b2342, b2012, b0895, b2659, b3092, b1218, b1000, b2579, b2537, b4122, b1947, b4021, b2341, b3541, b2463
